# Influence of serotonin on the long-term muscle contraction of the Kohnstamm phenomenon

**DOI:** 10.1038/s41598-025-00444-1

**Published:** 2025-05-13

**Authors:** Annika Schmidt, Tobias Meindl, Alin Albu-Schäffer, David W. Franklin, Philipp Stratmann

**Affiliations:** 1 TUM School of Computation, Information and Technology, Technical University of Munich (TUM), 85748 Garching, Germany; 2 Institute of Robotics and Mechtronics, German Aerospace Center (DLR), 82234 Wessling, Germany; 3 Munich Institute of Robotics and Machine Intelligence (MIRMI), Technical University of Munich (TUM), 80992 Munich, Germany; 4 Department of Neurology, University Hospital rechts der Isar, Technical University of Munich, 81675 Munich, Germany; 5 Neuromuscular Diagnostics, TUM School of Medicine and Health, Technical University of Munich, 80992 Munich, Germany; 6 Munich Data Science Institute (MDSI), Technical University of Munich, 80992 Munich, Germany

**Keywords:** Motor control, Excitability

## Abstract

Neuromodulation plays a central role in human movement control. An imbalance of neurotransmitters, especially dopamine and serotonin, can be associated with various neurological disorders causing tremors or spasms. Specifically, serotonin was shown to scale motoneuron excitability following intense muscle contractions, affecting short-latency reflexes. Likely, it may also influence motoneuron modulation in prolonged contractions, although this lacks experimental evidence. An intriguing test case for this hypothesis is presented by the Kohnstamm phenomenon, where sustained muscle contractions lead to prolonged amplified EMG activity and involuntary motions, aligning with the timescale of serotonergic amplification. The suspected serotonin influence on this effect was tested in a placebo-controlled human user study with 14 participants, where half were administered the serotonin antagonist Cyproheptadine and the other half a placebo. Comparing EMG and force responses after inducing the Kohnstamm phenomenon in the deltoid muscles revealed statistically significant faster EMG decay with the serotonin antagonist, while decay remained consistent in the placebo group compared to the response of the same participant group without medication. The force measurements showed the same trend, although no significance. This provides new data-based evidence that serotonin contributes to long-term motoneuron modulation, extending previous findings about the dedicated role and influence of this neurotransmitter. Additionally, the work suggests the phenomenon as an interesting test case to investigate the dedicated involvement of different neurocontrol mechanisms such as Persistent Inward Currents.

## Introduction

A central feature of human voluntary motion control is the ability to actively suppress involuntary movements^1,2^. Neurological disorders such as Parkinson’s disease or dystonia seem to impair this ability, causing uncontrollable movements that manifest in tremors or spasms^1,3^. These disorders are usually attributed to an imbalance in neurotransmitters, specifically dopamine and serotonin^4^. Consequently, both must play pivotal roles in motor control mechanisms. Indeed, recent studies have unveiled a subtype of dopamine neurons that appears to correlate with accelerations^5,6^, expanding beyond the well-established reward-related signaling^7^. Likewise, the raphe nucleus, releasing serotonin from the brain stem into the spinal cord^8^, was found to receive proprioceptive feedback^9^. Given the dense projection of the serotonergic system to spinal motoneurons^10,11^, it is believed to modulate various motor actions. Notably, Wei et al.^12^ experimentally demonstrated that serotonin diffusely scales motoneuron excitability following intense muscle contraction. It is likely that this observation in the context of sustained contractions is at least in parts caused by the modulation of the *Persistent Inward Currents* (PIC) through serotonin^13^. Upon further examination of the serotonergic feedback loop, it is suggested that this serotonergic gain scaling may not only have a diffuse impact but could also involve a topographically specific component^14,15^. Our previous experiments^16^ investigated this hypothesis and showed again that serotonin provides scaling of motoneuronal gains, and this scaling appeared to be adjusted in a joint-specific manner: repetitive, prolonged movement of one joint increased motoneuron excitability in its associated muscles, leaving others unaffected. While serotonin influence on PICs are likely involved in the excitability scaling of motoneurons of proximal joints after sustained contractions^17,18^, the exact role of the neuromodulator in the observed joint-specific effects during fast dynamic motions is still unclear^16^. Nevertheless, the neuromodulator remains the primary candidate for motoneuron gain adjustments following prior mechanical input.

The onset of the serotonergic amplification effect after sensory stimulation was observed to be delayed by several milliseconds to a few seconds^12,15^. Coincidentally, this timescale aligns with that of the Kohnstamm phenomenon, where a sustained muscle contraction of 30–45 s in a joint will result in the involuntarily lift of the same joint after a short latent period of 0.1–1 s ^1,19^. Thus, the sustained activation of the innervated joint muscles during this phenomenon fits the time scale observed for human serotonin levels to return to baseline^12^, suggesting serotonin’s involvement in the observed effect, likely through modulation of the PICs in the contracted muscles^19^.

Therefore, we designed an experiment to test the hypothesis that long-term motoneuron modulation through serotonin is observable during the Kohnstamm phenomenon. Fourteen participants were tested with medication-free morning experiments, while afternoon trials involved half of the participants taking the serotonin antagonist Cyproheptadine, with the other half receiving a placebo in a double-blind fashion. The serotonin antagonist was administered to the participants as a pill with a dose of 8 mg Cyproheptadine (tradename Peritol), as proposed by Wei et al.^12^.

All participants were unfamiliar with the Kohnstamm phenomenon and naïve to the experiment’s purpose, setting this study apart from most previous investigations on the Kohnstamm phenomenon^1,19,20^. During the experiments, the participants were positioned with their arms attached to a horizontally moving manipulandum to dissociate sensorimotor responses from gravitational effects. The Kohnstamm phenomenon was triggered in the deltoid muscle, and responses in the following relaxation period were quantified by measuring the muscle’s EMG signals and the projected force applied to the manipulandum. Both signals were normalized by measurements of initial MVC recordings to make them dimensionless and comparable between subjects. After validating the Kohnstamm phenomenon was triggered, two integral metrics were introduced to quantify the strength and duration of the triggered force ($F_I$) and the deltoid EMG ($EMG_I$) in a fixed time frame (0.2–2 s) after the induction period.

As hypothesized, the Cyproheptadine group showed weaker and shorter force and EMG signals in the afternoon compared to the morning, with the $EMG_I$ metric confirming a significant difference from the placebo group and indicating faster EMG decay. While $F_I$ differences were not statistically significant, observed trends aligned with the EMG findings, suggesting that the serotonin-antagonist weakened the Kohnstamm phenomenon.

This extends previous findings about the modulation of the short-latency stretch reflex through serotonin^12^. Serotonin accordingly seems to be also involved in controlling longer-lasting involuntary movements in healthy humans. This aligns with the observations in neurological disorders, where defective serotonergic functions seem to contribute to manifest involuntary motions^4,21,22^. Using the Kohnstamm phenomenon as a tool to further decipher the influence of serotonin and associated feedback loops in involuntary movements in healthy humans could benefit the development of more effective treatment methods for such neurological disorders.

## Results

Initially, it was validated that the Kohnstamm phenomenon was triggered in the experiment setup by identifying characteristic patterns expected for the observed EMG and force measurements. Following, the influence of serotonin was quantified with the introduced integral metrics for force ($F_I$) and the deltoid EMG ($EMG_I$), and the decay times of the signals after the induction period were compared.

### Verification of the Kohnstamm phenomenon

Usually, the most obvious effect of the Kohnstamm phenomenon is the sustained muscle activation after the induction period creating a force that leads to an observable involuntary movement. Thus, in many experiments, the strength of the Kohnstamm phenomenon is quantified by the resulting joint motion, but this is also believed to act as an additional feedback loop influencing the effect^19^. To exclude this feedback influence, the arm motion in this study was purposefully blocked during the relaxation period. Thus, the resulting muscle activation and force measurements were expected to result directly from the afferent input during the induction period and should reflect the known characteristics of the Kohnstamm phenomenon ^23^.

**Fig. 1:**
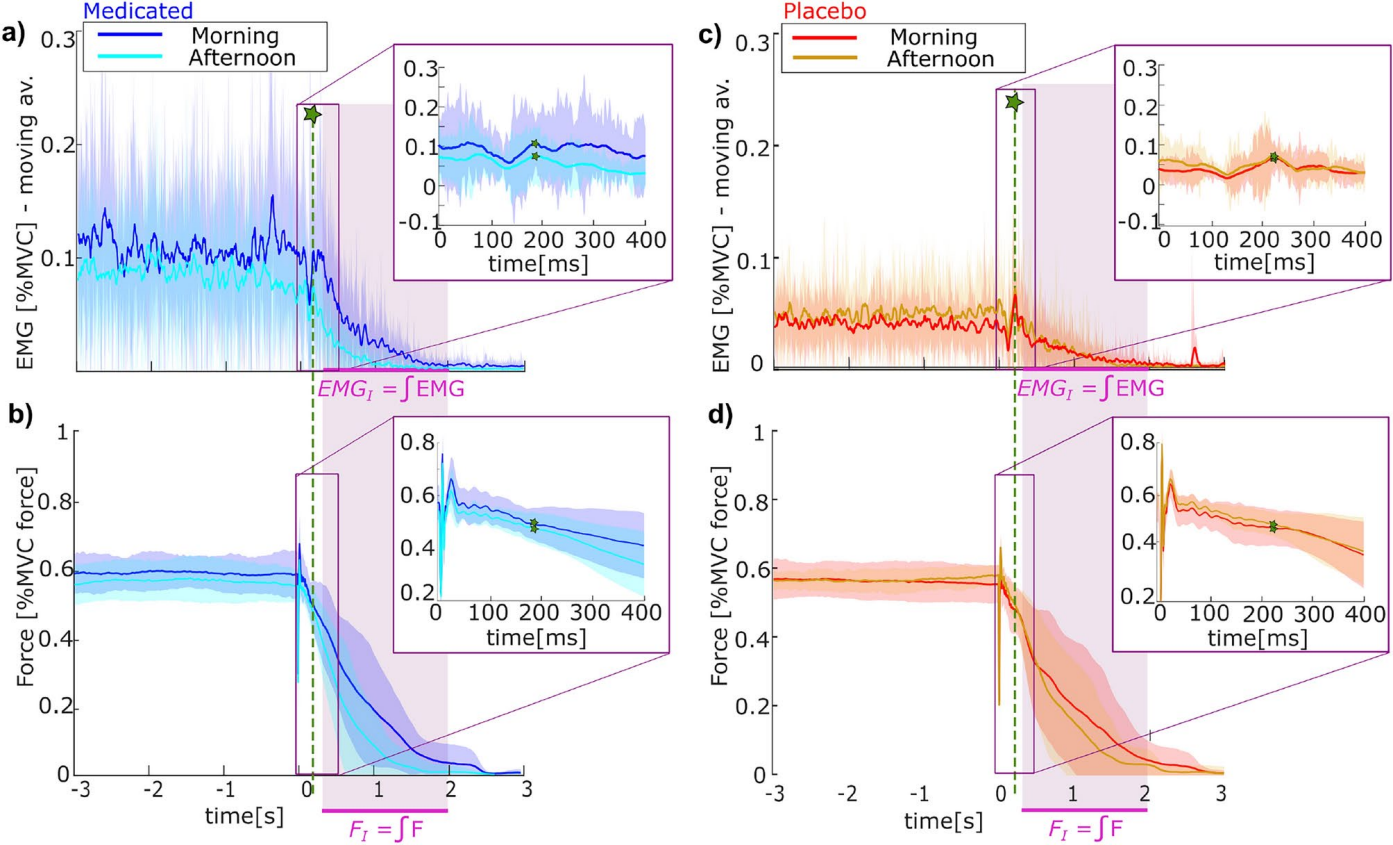
Measurement progression during the Kohnstamm phenomenon for the (**a, c**) sliding mean of the deltoid EMG signals with a window of $w=100$ ms and (**b, d**) force applied by the participants to the manipulandum handle. The shown measurements are averaged values over the three trials of the participants in the test group (n=7, left) and control group (n=7, right) for the morning and afternoon experiments, respectively. The error bands indicate the variability between the participants. The force and EMG values are normalized by the respective recordings of the MVC measurement taken prior to the experiments in the morning. The magenta-colored patch indicates the time span over which the integral metrics $F_I$ and $EMG_I$ were calculated, where $t=0$ s indicates the end of the induction period, which was immediately followed by a relaxation period of the participants. For each comparative plot, a zoom of the measurements over the time span of $t=0:400$ ms is presented, in which the drop in the EMG signals marks the latent period that is expected in the Kohnstamm phenomenon. The green star marks the end of the latent period, where the EMG signal increased again. From this point, the decay rate of the different signals was analyzed as depicted in Fig. 2.

Indeed, the onset of the relaxation is apparent in the force plots (Fig. 1b,d), where an immediate jump at $t=0$ s indicates the end of the induction period since the participants’ reaction was slightly delayed. The peak is followed by a force plateau when the participant starts to relax and stops consciously applying force. Subsequently, the force continues to drop during the relaxation period until it reaches zero after around 3 s. Simultaneously to the force progression, a short drop in the EMG signal can be observed (Fig. 1a, c; zoom). This characterizes the latent period typical for the Kohnstamm phenomenon. Additionally, some participants proactively reported an odd feeling of lightness accompanying the onset of the relaxation. On average, the mean time duration until the EMG signal of the participant reached its rest level in the relaxation period was 1 s ± 0.6 s with the handle force subsiding in the same time frame.

Consequently, the participant measurements support the expectation that the Kohnstamm phenomenon was indeed triggered in the investigated experiments, even though participants were unaware of the expected effects. This sets the stage for a deeper analysis of the specific influence of serotonin in the following.

### Influence of serotonin

As hypothesized, the experiment revealed significant differences between the two participant groups that suggest an involvement of serotonin in the control of the Kohnstamm phenomenon.

When first comparing the morning and afternoon measurements individually per participant group, it appears that in the test group taking Cyproheptadine, the EMG signal is offset in the afternoon from the morning measurements (Fig. 1a), while in the control group the EMG drop appears consistent (Fig. 1c). The force applied to the manipulandum handle after the induction period appears to drop faster in the afternoon for both groups (Fig. 1b, d). However, a quantitative comparison is difficult since it appears that the participants of the test group generally needed higher muscle contractions to realize the required force during the induction period. While the participants taking Cyproheptadine applied $10\%$ of the MVC EMG signal (Fig. 1a), the participants of the control group only needed around $5\%$ of the MVC EMG signal (Fig. 1c). This general observation of the averaged EMG data is reflected in the individual data, which suggests different physical capabilities and muscle contractions of the different groups, making a comparison between the groups non-trivial.

**Fig. 2:**
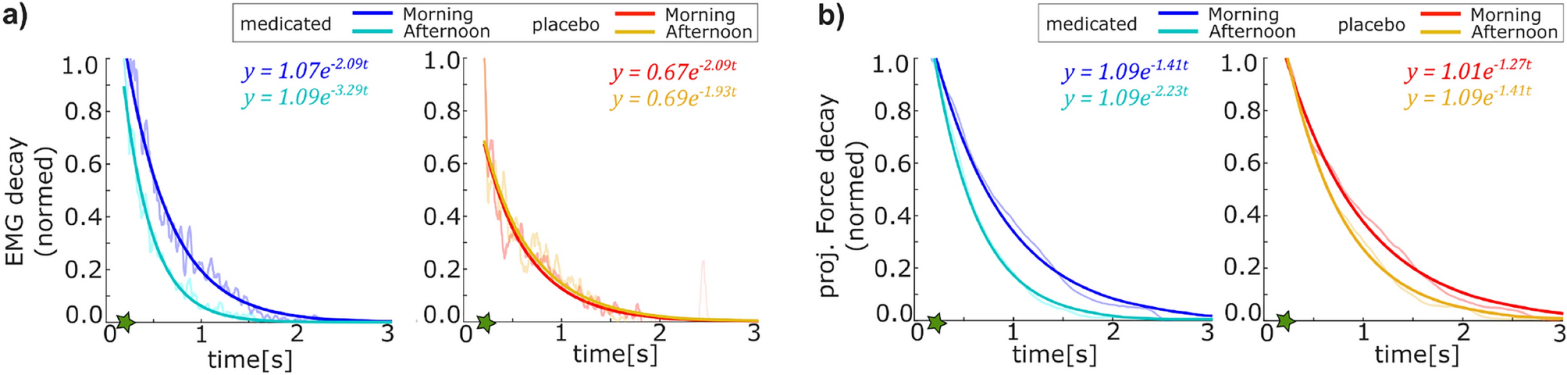
Signal decay after the latent period of the Kohnstamm phenomenon of the (**a**) EMG measurements of the deltoid muscle and (**b**) applied handle force. The respective measurements were normalized relative to the values that were obtained after the latent period (marked with a green star, see also Fig. 1a,c). The averaged data points for all participants in each group per condition (n=7) were fitted with a curve of the form $y = A_0 e^{\lambda t}$. Determined parameter values are stated in the respective plots.

To account for this effect and enable the comparison of the different groups, the decay rate of the normalized EMG and force signals was regarded. Considering the peak value after the EMG drop during the latent period as the starting point (Fig. 1, green star), each signal was divided by the respective EMG and force value of this point such that signals were normalized to 1. Regarding the decay from this normalized value, until zero is reached, we can quantify how fast the EMG and force signals dropped for each group in each condition, leading to comparable measures. As depicted in Fig. 2, this comparison confirmed the initial impression that the EMG signal of the test group receiving Cyproheptadine decayed much faster in the afternoon compared to the morning. In contrast, the control group taking the placebo did not exhibit this increased EMG decay but appeared consistent with the morning measurements (Fig. 2a). The decay rate of the force in the normalized comparison appears greater in the afternoon for both groups, however, the drop difference compared to the morning measurements is more pronounced in the test group taking Cyproheptadine (Fig. 2b).

**Fig. 3:**
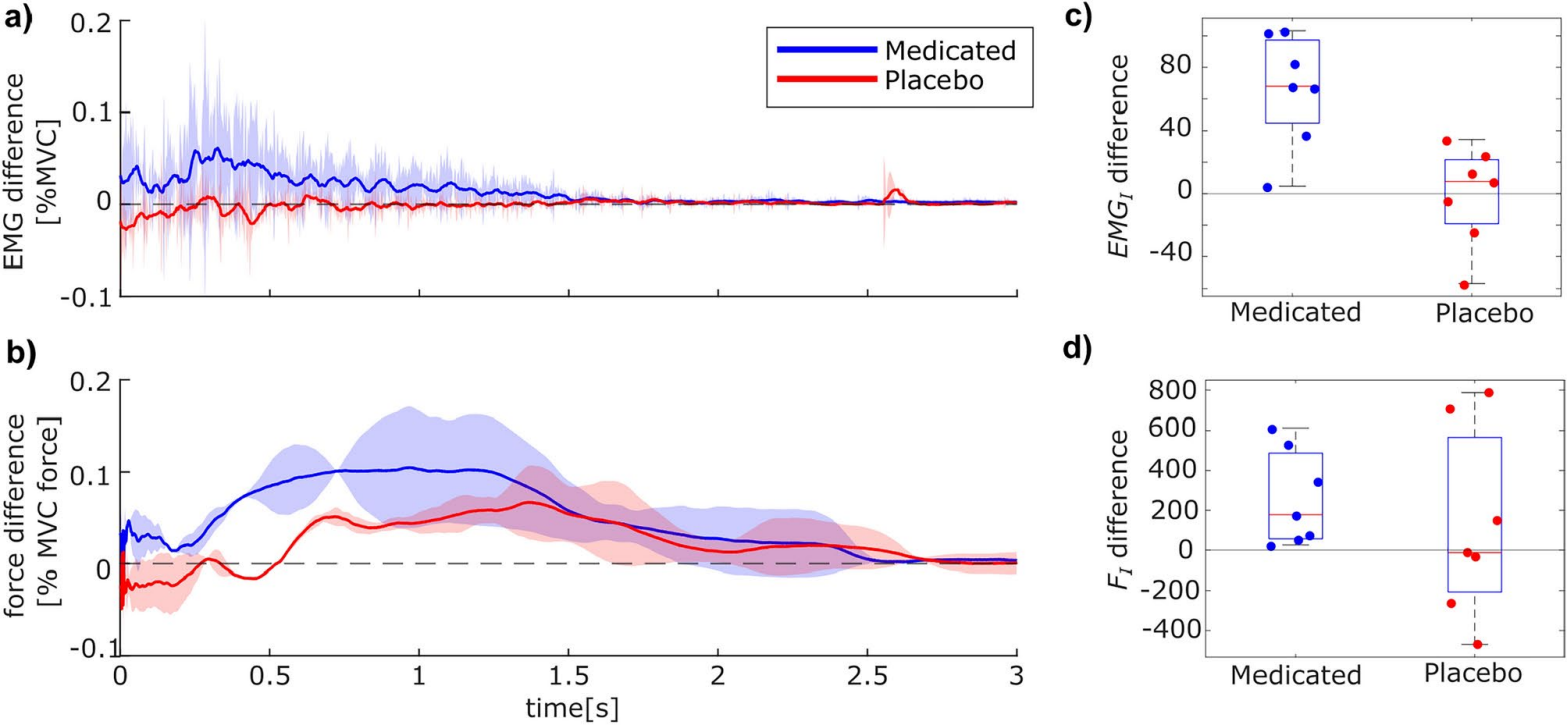
Difference of morning and afternoon trials for both participant groups. Differences of the averaged measurements of the test group taking Cyproheptadine ($n=7$, blue) and the placebo control group ($n=7$, red) were obtained by subtracting the average afternoon values from the morning values for (**a**) the EMG measurements of the deltoid and (**b**) the force applied by the participants to the manipulandum handle. The solid lines of the force measurements show the average over all trials per participant group, while the patches visualize the standard deviation of each group. For the EMG measurements, the solid lines indicate the sliding mean of the signal with a window of $w=100$ ms. The measurements are plotted for the first three seconds after the onset of the relaxation period, where the Kohnstamm phenomenon should be triggered. (**c**) Inspecting the values differences for $EMG_I$ likewise suggests that the triggered muscle contractions in the afternoon were identical to the morning for the control group but lower in the test group. (**d**) The difference of the integral metric $F_I$ between the morning and afternoon trials suggests that the average difference for the control group was around zero. In contrast, the test group showed overall reduced values in the afternoon.

A closer examination of these observations provides visual support for this notion, illustrated by plotting the differences between the morning and afternoon measurements (Fig. 3). The force drop appears more pronounced in the test group, and the positive EMG signal difference suggests that the average muscle contraction signal was weaker in the afternoon. In contrast, the average difference of the EMG signal for the control group taking the placebo appears close to zero (Fig. 3a). Regarding the introduced integral metrics $F_I$ and $EMG_I$ shows a similar trend: comparing the resulting integral of the force signal shown in Fig. 1b, d over the time period of $t = 0.2:2$ s for the morning and afternoon trials suggests a average difference around zero for the control group (Fig. 3d, red). Contrastingly, the difference of $F_I$ between the morning and afternoon trials appears larger than zero for the test group (Fig. 3d, blue). This observation seems even more pronounced for the integral of the EMG signals (Fig. 3c). However, it must be acknowledged that the spread of the individual participant values is large throughout. Nevertheless, the individual participant values for the $F_I$ and $EMG_I$ differences show a clear trend of being larger than zero compared to the control group. This indicates that taking Cyproheptadine could noticeably weaken the strength and duration of the force and EMG signals triggered during the Kohnstamm phenomenon.

To investigate this hypothesis statistically, a linear-mixed-effect model was applied to the data detailed in (3). This takes into account the morning trials, which all participants carried out soberly, as well as the afternoon trials, where either the placebo or Cyproheptadine was administered. In the linear mixed-effect model, the fixed-effect size $\beta _\textrm{1}$ describes by how much the response of an individual subject (i.e. their $EMG_I$ or $F_I$) value changed if they performed the experiment before any intake against after Cyproheptadine intake; or in the hypothetical case that the subject would have received a placebo instead of Cyproheptadine. To account for the difference in starting EMG and force levels prior to the Kohnstamm phenomenon onset between the groups, these values were included as covariates. Comparing the EMG-based metric $EMG_I$ before and after drug administration shows that Cyproheptadine significantly reduced the Kohnstamm effect with $p = 0.001$ ($df = 80, t = 3.36$). This effect is stable against the placebo control, as the Cyproheptadine group showed a Kohnstamm effect that was reduced by an additional effect size of $\beta _\textrm{1} = 22.92 \pm 10.58$ compared with the placebo control group at $p = 0.03$ ($df = 80, t = 2.17$). For the force metric $F_I$, Cyproheptadine also decreased the Kohnstamm effect by $\beta _\textrm{1} = 202.62 \pm 56.95$ with $p = 0.0006$ ($df = 80, t = 3.56$) in comparison to the trials before administration. While Cyproheptadine also decreased the Kohnstamm effect by $\beta _\textrm{1} = 144.31 \pm 77.21$ in comparison to the placebo control group, this difference was non-significant at $p = 0.065$ ($df = 80, t = 1.87$). This could, in part, be due to the large standard deviation, as shown in Fig. 3d (red). All relevant statistical values are presented in Table 1.

**Table 1:** Statistical values for the metric comparison of the medicated test group and the placebo control group analyzed with the linear-mixed-effect model.

Condition	$\pmb {EMG_I}$					$\pmb {F_I}$				
	Estimate	SE	tStat	DF	*p*-value	Estimate	SE	tStat	DF	*p*-value
vs. placebo	22.92	10.58	2.17	80	**0.03**	144.31	77.21	1.87	80	0.065
vs. morning	26.96	8.02	3.36	80	** 0.001**	202.62	56.95	3.56	80	**0.0006**

Significant values are in bold.

To ensure that the observed differences triggered by the Kohnstamm phenomenon were not caused by fundamental discrepancies during the induction periods, the applied forces of these periods per participant were compared. It was found that the participants of the medicated test group applied on average $57 \pm 8.6$ % of the MVC force during the 45 s long induction period to trigger the Kohnstamm phenomenon. With this, they fulfilled the requirement of applying around 60 % of the MVC force to trigger the Kohnstamm phenomenon. In the afternoon trials, the force applied during the induction period was with an average of $53 \pm 10.0$ % of the MVC force slightly smaller (Fig. 1b), but showed no significant difference over the test group participants when compared with a t-test. Similarly, the control group applied on average $56 \pm 5.3$ % and $57 \pm 2.2$ % in the morning and afternoon during the induction period, respectively (Fig. 1d). Again, the t-test reveals no significant difference. Thus, it can be assumed all participants applied force within the same range across morning and afternoon trials, ensuring consistent trigger conditions for the Kohnstamm phenomenon. Hence, the force and EMG responses in both runs should have been similar, as seen in the control group, indicating the differences in the test group were related to the serotonin antagonist.

Summarizing all reported findings, the intake of Cyproheptadine as a serotonin antagonist seems to cause a lower EMG response triggered through the Kohnstamm phenomenon. Although the statistical comparison of the force does not show a significant difference for the small sample size, the visual force differences of the morning and afternoon trials show a similar trend. Thus, the findings support our hypothesis that serotonin might be involved in the control of the Kohnstamm phenomenon and that administering the antagonistic medication changes the participants’ responses.

## Discussion

We carried out a human subject experiment to validate the hypothesis that serotonin is involved in the long-term motoneuron modulation observed in the Kohnstamm phenomenon. The study showed that participants who were administered the serotonin-antagonistic medication Cyproheptadine had weaker and shorter deltoid muscle contractions following the induction period in comparison to the same participants without the medication. This was supported through an EMG-based metric $EMG_I$ summarizing the strength and duration of the recorded EMG signals, which showed a significant difference to a placebo-taking control group. The analog force-based metric $F_I$, evaluating the exerted participant force on a manipulandum handle, showed a statistically significant difference between trials performed soberly and after Cyproheptadine administration, but failed to show a statistical difference between Cyproheptadine and placebo administration at $p=0.065$. Still, it suggested the same trend and direction of the fixed-effect regression coefficient $\beta _\textrm{1}$. The observation that this metric did not become significant could be associated with the fact that the forces were measured on the end effector, where the human participant was coupled to the manipulandum. The exerted forces in this point cannot be isolated to arise from one muscle only but are likely a contribution of multiple muscle contractions. It was, therefore, expected that the associated force metric would show less clear differences than the EMG metric, where the deltoid muscle was the only contributing factor. The statistically significant results for the EMG metric underline the involvement of serotonin in the modulation of the Kohnstamm phenomenon.

With these findings, our study provides data-based evidence supporting that serotonin is involved in the modulation of long-term muscle contractions. This expands the findings of Wei et al.^12^ about the amplification of the short-latency stretch reflex observed several hundred milliseconds after strong proprioceptive input. Although the underlying mechanisms involved in triggering the Kohnstamm phenomenon are not yet fully understood, the observed effects of serotonin on the aftercontraction could suggest that Persistent Inward Currents (PICs) may be one of the driving mechanisms. These currents in motoneurons result from activation of the brain stem serotonergic and noradrenergic systems^17,24^ and are thus strongly modulated through these neurotransmitters^25^. Consequently, the initial voluntary contraction during the induction period likely activates the PICs^19^, which are known to enable motoneurons to continue firing even after the excitatory input has stopped^18,26^ leading to the increased force and EMG production in the relaxation period. Additionally, PICs deactivate slowly, which matches the gradual decay observed in the Kohnstamm phenomenon. The administration of the serotonin antagonist in the presented study can thus be expected to decrease the strength of the activated PIC, leading to a weaker response, as observed in the test group in the afternoon trials. Although it is likely that other mechanisms are also involved in the modulation of the Kohnstamm phenomenon, the activation through PICs aligns well with the observations from our experiments. Although the effects were only investigated in a single muscle, it can be expected that the results are reproducible in other joints since early studies have already shown that the Kohnstamm phenomenon can be elicited in multiple joints of the arms and legs^27,28^. The phenomenon seems to be especially strong in proximal joints innervated by large muscle groups^19,29^, which further supports the suspected involvement of PICs known to be stronger in proximal muscles^13^. Thus, the Kohnstamm phenomenon might be an interesting case to investigate the role of PICs in voluntary and involuntary movements specifically, as it offers a simple setup where both movement forms occur^30^. Taken together with our previous study on joint-specific gain scaling^16^, the findings stress the fundamental importance of serotonin for motor adaptation. The monoamine acts at just the right time scale to react to quick changes while accumulating information throughout a movement.

However, the limitations of our study should also be reviewed critically: The group participating in the experiment was small, with only seven individuals per group, and only the right deltoid muscle was investigated. The induction period was controlled for force with the expectation that relative to the individual MVCs, the needed EMG magnitude to produce this force should be similar for all participants. However, analyzing the experiment data revealed that all participants of the control group taking the placebo elicited smaller EMG signals to produce the same force relative to their MVC force. This can most likely be attributed to random individual differences between the participants who received Cyproheptadine or the placebo. For example, participants in the control group might have a different motor unit recruitment pattern and recruit larger, more efficient motor units earlier; or they may have a different muscle fiber composition, with a higher proportion of type I / slow-twitch fibers that need lower EMG activity over sustained submaximal efforts. Since the study was carried out double-blind, such features could not be accounted for by systematically grouping the participants. Nevertheless, the lower EMG signals in the participants of the control group were consistent in the morning and afternoon trials, which indicated that the observed difference in EMG magnitude between the two groups can indeed be attributed to individual body composition. Still, the study results should be validated with a bigger cohort, where EMG measurements from antagonistic muscles should also be taken to better control the observed force production and account for effects like reciprocal inhibition.

Nevertheless, it is noteworthy that despite the limited number of participants, significant differences between the test group and control group could be found in the tested deltoid muscle. This suggests that Cyproheptadine and the associated modulation of neurotransmitters indeed have an influence on the manifestation of the Kohnstamm phenomenon. However, it is important to acknowledge that Cyproheptadine affects not only serotonin levels but also exerts a strong impact on histaminergic pathways. Although no studies have specifically explored the role of histamine in the Kohnstamm phenomenon, its influence is plausible since a blockage of the H1 receptors could potentially enhance proprioceptive feedback from muscle spindles, which may contribute to postural aftercontractions^31^. However, H1 receptors are primarily involved in regulating muscle spindle activity^32^, which requires proprioceptive input to be effective. In the reported experimental setup, the arm motion during the aftercontraction was blocked, which minimized proprioceptive feedback. Thus, it is more likely that the observed effects of the presented results are related to changed motoneuron excitability modulated through serotonin^16^, e.g., through the activation of PICs. Nevertheless, the potential modulation of the Kohnstamm phenomenon by both histamine and serotonin is plausible, and their distinct roles should be explored in future studies.

Additionally, it needs to be noted that the arm motion was fully blocked by the high friction of the manipulandum device in the investigated experiment setup. This friction was purposefully not compensated to exclude the hypothesized modulation of the aftercontraction through position feedback^19^ and isolate the effect caused by the afferent input during the induction period. Thus, the observed time frame of the aftercontraction of around 1 s was shorter than the typically observed duration in the unobstructed experiment setting, where multiple seconds of aftercontraction have been reported^33^. Although the observed time frame in our experiment was short, it was still considerably longer than expected after a voluntary moderate contraction, where it is assumed that EMG signals return to rest within 100–200 ms^34,35^. This observation, paired with the short latent period identified in the first 200 ms for most trials and the recorded forces despite no active force application after the induction period, suggests that the Kohnstamm phenomenon was indeed triggered in our experiments and the observed effects can be attributed to the long-term modulation of muscle signals. This supports other previous research suggesting that the Kohnstamm phenomenon must be partly controlled through central control mechanisms, like PICs, if its effects are observable when motions are completely obstructed^23^. In fact, this central feature could be attributed to the release of serotonin, as already discussed. Nevertheless, it can be assumed that the effects would have been more distinct in a setup where the arm could move freely.

In summary, the presented study provided new data-based evidence aligning with previous research aiming to decipher the role of serotonin in the control and modulation of motoneuron signals. Validating the involvement of serotonin in the long-lasting muscle contractions triggered by the Kohnstamm phenomenon presents it as a prime example case to investigate the functioning and role of this neurotransmitter further. Considering this next to the more traditional investigations of the short-latency stretch reflex might reveal new insights about the motoneuron modulation through serotonin. In turn, this could contribute to developing new treatment methods for neurological disorders associated with neurotransmitter imbalances, such as Parkinson’s disease.

## Methods

### Ethics statement

The study was approved by the Ethics Committee of the Medical Faculty of the Technical University of Munich and was carried out according to the principles expressed in the Declaration of Helsinki. Participants had to be right-handed and have no known impairments of the neuromuscular or musculoskeletal system. Prior to the experiment, all participants underwent a targeted physical examination by a medical doctor to ensure no contraindications against the administration of Cyproheptadine existed. All participants provided written informed consent before participating.

### Participants

The experiment was carried out by 14 right-handed participants (4 female, 10 male, on average 25.6 years). All were all naïve to the purpose of the experiment and reported after the experiment that they either had never heard of the Kohnstamm phenomenon or were unaware that it was being investigated. The participants were randomly divided into two groups: (1) a control group that was given a placebo and (2) a test group that was administered Cyproheptadine, which acts on metabotropic serotonergic receptors of motoneurons^12^. The placebo pills were of color and dimension identical to the chosen serotonin antagonist (white, 8-mm diameter; produced by Zentiva). They contained no active substance, solely consisting of filling material, namely lactose monohydrate, cellulose powder, magnesium stearate, and microcrystalline cellulose. The study was performed double-blind such that neither the participant nor the experimenter knew to which group the participant belonged. As an individual reference, all participants carried out the experiment in the morning without medication. The experiment was then repeated in the afternoon after the administration of the placebo and Cyproheptadine, respectively.

### Experimental setup

**Fig. 4:**
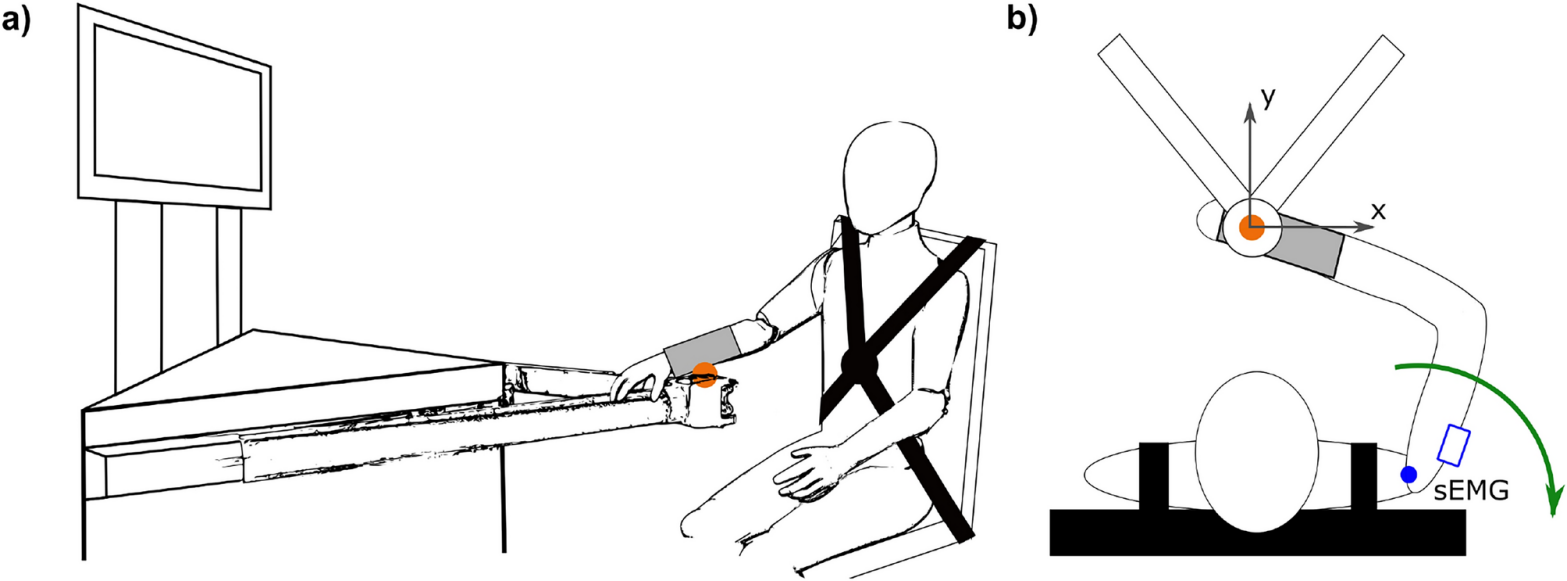
Experimental setup. (**a**) Perspective sketch of the experiment setup, in which the participant’s wrist is connected to the handle of a custom-built manipulandum by means of a splint (orange). (**b**) The position of the shoulder joint can be tracked through a goniometer, and the muscle activity of the posterior deltoid is recorded by a sEMG electrode (blue). The position and exerted forces of the manipulandum handle (orange) can also be measured. During the experiment, the participant is tasked to pull the shoulder backward (green) while the manipulandum handle is commanded to stay in a fixed position.

In the experiment setup, the participant was fixated in a chair with the right arm being attached to a manipulandum through a splint, as shown in Fig. 4a and b (orange). The height of the participant’s seating was adjusted such that the arm attached to the manipulandum moved in a horizontal plane. The upper arm was supported by a strap against gravity to reduce fatigue in the participants. Therefore, the Kohnstamm phenomenon and its effects were investigated independently of gravitational influences. A screen in front of the participants visualized task instructions during the experiment. The position of the manipulandum end effector, i.e., the attachment point with the human arm, was commanded through two linear actuators, which were equipped with corresponding sensors to measure the position in Cartesian space. The forces that both participants and the linear motors exerted onto the attachment point were measured by a six-axis force-torque sensor (*mini45*, *ATI Industrial Automation*, USA). To measure the participant’s muscle activation, a wireless surface EMG electrode (*Trigno Avanti*, *Delsys*, USA) was attached to the skin over the posterior deltoid, chosen according to recommendations by the *SENIAM* project^36^. Additionally, a goniometer (*SG150B*, *Biometrics*, USA) was attached over the shoulder joint to track the angle of the joint (Fig. 4b, blue). The intrinsic recording delay was 48 ms in the EMG electrodes and 96 ms in the accelerometers and goniometers. The carried out analysis corrected for all delays. All signals were sampled at 2 kHz.

### Experimental procedure

Prior to the experiments, the Maximum Voluntary Contraction (MVC) of the deltoid muscle of each participant was measured as an individual reference point. For this, the participants were asked to pull the shoulder backward (Fig. 4b, green) while the attachment point to the manipulandum was controlled to be fixed in position. In addition to the EMG measurement, the exerted force on the handle was recorded. This procedure was repeated three times, and the EMG and force averages of these trials were taken as individual references.

For the experiment measurements, the participants were asked to once more pull the shoulder backward to exert a force on the attachment handle that was $60\%$ of the force applied during the MVC measurements. The handle was again controlled to remain fixed so the participant could pull against it. To sustain a constant effort, the screen in front of the participant showed a bar that was scaled to the applied force and should be kept in a defined shaded area (50–70%$F_{MVC}$). This force effort had to be maintained for 45 s, counting down onscreen. A click indicated the end of this induction period, after which the participant had been instructed to completely relax the arm. The controller fixing the handle in place was released in the relaxation period as the remaining manipulandum friction was sufficient to oppose passive motions and allow clean force measurements. Thus, an active motion of the manipulandum handle during this period indicated that the participants were not relaxing, and the trial was repeated. The muscle signals of the relaxing participants were recorded for 30 s. Following, another 30 s was given to the participant to prepare for the next trial of this experiment. Three trials were carried out in the morning without medication, as well as in the afternoon after administration of either a placebo or Cyproheptadine.

### Metrics and statistical analysis

**Fig. 5:**
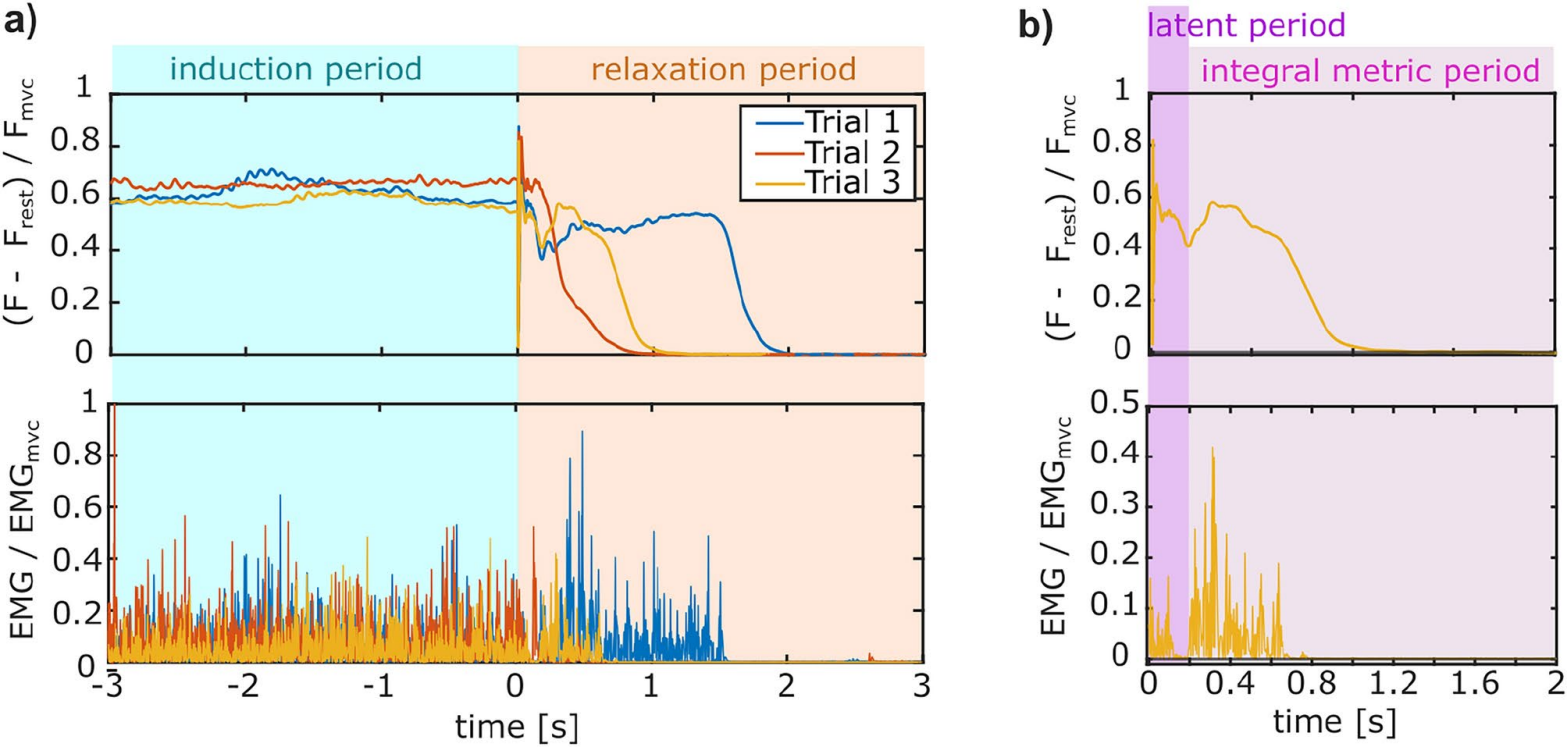
Outline of measurement analysis. (**a**) Recorded measurements of the force *F* applied to the manipulandum handle (top) and corresponding EMG signals (bottom) over the three morning trials exemplary shown for participant 1. The time $t=0$ s marks the end of the induction period, where the participants had to continuously exert force for 45 s to trigger the Kohnstamm phenomenon. (**b**) Zoom in on the measurements of trial 3 to indicate the latent period marked by the drop in the EMG measurements. Over the marked period of $t=0.2:2$ s, the metrics $EMG_I$ and $F_I$ were calculated to quantify the strength and duration of the induced force and muscle contractions.

All data was analyzed with Matlab2020b. To investigate the influence of serotonin on the Kohnstamm phenomenon, two main measurement recordings were chosen based on characteristics known to be triggered in the phenomenon, i.e., sustained muscle activation resulting in force generation moving the involved joints if not obstructed. Thus, the first important metric was the EMG measurements of the deltoid muscle, which had to be contracted in the induction period. Since the arm motion was purposefully obstructed through the high friction forces of the manipulandum to avoid influences through position feedback, additionally, the exerted force *F* on the handle was analyzed.

For the comparable analysis of these measurements, all EMG measurements were rectified and normalized by the MVC measurements recorded before the morning experiment. Likewise, the force measurements were normalized by the corresponding average force during the MVC recordings after subtracting the resting force measured in a relaxed state without any force exertion. Thus, all the following analyses were carried out using the relative dimensionless data. Exemplary, Fig. 5a shows the pre-processed data for the three trials of Participant 1, and Fig. 5b shows a zoom-in on the third trial.

Despite the rectification, the EMG comparison of the participants of the test group taking Cyproheptadine and the control group taking a placebo suggested fundamental physical differences between the participants of the different groups. To achieve the same force in the induction period, the test group applied $10\%$ of the EMG contraction measured during the MVC condition, while the control group only applied an averaged $5\%$ of the MVC EMG. In order to still allow a fair comparison between the two groups, the averaged EMG and force measurements were also normalized by the value observed in the first peak after the latent period (Fig. 1, green star). Thus, all normalized data signals decay from an initial value of 1. These normalized signals were fitted with the built-in Matlab function fit function using a handle describing an exponential decay, which was suggested by the data

1 $$\begin{aligned} y(t) = A_0 \ e^{-\lambda t} \ , \end{aligned}$$

where the exponent $\lambda$ quantified how fast the error signal decayed. Showing that the observations of the rectified data reflected in the normalized signals justified further comparisons between the test group and the control group.

Therefore, For each of the two investigated measurements, i.e., force and EMG, a metric was defined to quantify the effects induced through the Kohnstamm phenomenon. These metrics, namely $EMG_I$ and $F_I$, were chosen to be the integral of the respective measurements taken for a defined time period (Fig. 5b, magenta), such that it captured the strength of the induced motoneuronal effects as well as the duration:

2 $$\begin{aligned} EMG_I = \int _{t_1}^{t_2} \frac{EMG_{\textrm{deltoid}}}{EMG_{\textrm{MVC}}} \textrm{d}t; \qquad F_I = \int _{t_1}^{t_2} \frac{F_{handle}}{F_{MVC}} \textrm{d}t; \end{aligned}$$

The investigated time frame was set to be from $t_1 = 0.2$ to $t_2 = 2$s (Fig. 5b). The time period of 2 s was considered sufficiently long, as the data showed that the triggered effects had ceased after 3 s. At this point, the EMG signals returned to the rest EMG and no more forces were applied to the manipulandum handle. The time span until 0.2 s was neglected for the metric calculation, as this included the delayed onset of the participants’ relaxation and the latent time of the EMG signal expected in the Kohnstamm phenomenon^1,19^. In order to norm the metrics that were integrated over 1.8 s, the resulting metric values were divided by this factor. To compare the EMG and force metrics statistically, a linear mixed-effect model was used:

3 $$\begin{aligned} r_i = \beta _\textrm{0} + \beta _\textrm{1} \cdot \textrm{med}_\textrm{i} + \beta _\textrm{2} \cdot \textrm{signal}_\textrm{init,i} + b_\textrm{0,m} + \epsilon _\textrm{im}\ , \end{aligned}$$

where $\beta _\textrm{0}$, $\beta _\textrm{1}$ and $\beta _\textrm{2}$ denote the fixed-effect regression coefficients, $b_\textrm{0,m}$ is the random-effect regression coefficient, and $\epsilon _\textrm{im}$ denotes the residuals. The subjects were denoted by *m*, and differences between them were considered as random effects. Due to the apparent differences, especially in the applied EMG value during the induction period between the participants of the test and control group, the starting EMG and force values $\textrm{signal}_\textrm{init,i}$ meaned over the last 4 s prior to the beginning of the Kohnstamm onset were included as a covariate. The fixed effect $\textrm{med}_i$ describes the medication taken for the respective measurement as

4 $$\begin{aligned} \textrm{med}_\textrm{i} = {\left\{ \begin{array}{ll} 1 & \text {if Cyproheptadine} ,\\ 2 & \text {if Placebo} ,\\ 3 & \text {if no intake (morning)} . \end{array}\right. } \end{aligned}$$

Since the hypothesis is that serotonin is involved in the motoneuronal control of the Kohnstamm phenomenon, it is assumed that the administration of the serotonin-antagonist Cyproheptadine will observably alter the firing activity of the motoneurons in the deltoid. Consequently, the EMG measurements of the test group participants are expected to be significantly different from the morning measurements (condition 3) as well as from the placebo group. The corresponding null hypothesis $\beta _{\textrm{0}}$ = 0 was tested by a two-tailed t-test.

To ensure that the force applied to induce the Kohnstamm phenomenon was constant for all participants in the morning and afternoon trials, the individual average difference of the force applied during the induction period in the two experiment runs was tested against zero with a t-test ($\alpha =5$ %).

## Data Availability

The raw data of the experiments is available on *figshare* under https://doi.org/10.6084/m9.figshare.27604974. One data folder is provided per subject, each including the measurements of the morning and afternoon sessions.
